# Manual hyperinflation partly prevents reductions of functional residual capacity in cardiac surgical patients - a randomized controlled trial

**DOI:** 10.1186/cc10340

**Published:** 2011-08-05

**Authors:** Frederique Paulus, Denise P Veelo, Selma B de Nijs, Ludo FM Beenen, Paul Bresser, Bas AJM de Mol, Jan M Binnekade, Marcus J Schultz

**Affiliations:** 1 Department of Intensive Care Medicine, Academic Medical Center, Meibergdreef 9, 1105 AZ, Amsterdam, The Netherlands; 2 Department of Anesthesiology, Academic Medical Center, Meibergdreef 9, 1105 AZ, Amsterdam, The Netherlands; 3 Department of Respiratory Medicine, Academic Medical Center, Meibergdreef 9, 1105 AZ, Amsterdam, The Netherlands; 4 Department of Radiology, Academic Medical Center, Meibergdreef 9, 1105 AZ, Amsterdam, The Netherlands; 5 Department of Respiratory Medicine, Onze Lieve Vrouwe Gasthuis, Oosterpark 9 1091 AC, Amsterdam, The Netherlands; 6 Department of Cardiothoracic Surgery, Academic Medical Center, Meibergdreef 9, 1105 AZ, Amsterdam, The Netherlands

## Abstract

**Introduction:**

Cardiac surgery is associated with post-operative reductions of functional residual capacity (FRC). Manual hyperinflation (MH) aims to prevent airway plugging, and as such could prevent the reduction of FRC after surgery. The main purpose of this study was to determine the effect of MH on post-operative FRC of cardiac surgical patients.

**Methods:**

This was a randomized controlled trial of patients after elective coronary artery bypass graft and/or valve surgery admitted to the intensive care unit (ICU) of a university hospital. Patients were randomly assigned to a "routine MH group" (MH was performed within 30 minutes after admission to the ICU and every 6 hours thereafter, and before tracheal extubation), or a "control group" (MH was performed only if perceptible (audible) sputum was present in the larger airways causing problems with mechanical ventilation, or if oxygen saturation (SpO_2_) dropped below 92%). The primary endpoint was the reduction of FRC from the day before cardiac surgery to one, three, and five days after tracheal extubation. Secondary endpoints were SpO_2 _(at similar time points) and chest radiograph abnormalities, including atelectasis (at three days after tracheal extubation).

**Results:**

A total of 100 patients were enrolled. Patients in the routine MH group showed a decrease of FRC on the first post-operative day to 71% of the pre-operative value, versus 57% in the control group (*P *= 0.002). Differences in FRC became less prominent over time; differences between the two study groups were no longer statistically significant at Day 5. There were no differences in SpO_2 _between the study groups. Chest radiographs showed more abnormalities (merely atelectasis) in the control group compared to patients in the routine MH group (*P *= 0.002).

**Conclusions:**

MH partly prevents the reduction of FRC in the first post-operative days after cardiac surgery.

**Trial registration:**

Netherlands Trial Register (NTR): NTR1384. http://www.trialregister.nl

## Introduction

Pulmonary dysfunction is a ubiquitous consequence of cardiac surgery [1]. Alterations in mechanical properties of the lung lead to reductions in pulmonary compliance [2] as well as vital and functional residual capacity (FRC) [3,4] in the first days after surgery. In addition, these patients remain sedated and are nursed in a supine position for several hours, while they are re-warmed and weaned from mechanically ventilation. This may reduce mucociliary transport, and as such retention of sputum [5,6]

In our setting, a frequently performed intervention as part of airway management of intubated and mechanically ventilated patients is manual hyperinflation (MH) [7-9]. MH aims at mobilization of retained airway secretions in intubated and mechanically ventilated patients, thereby preventing atelectasis [10,11]. With MH the patient is disconnected from the mechanical ventilator, after which the lungs are inflated via a resuscitation bag. The technique consists of several factors, including the application of a larger than normal breath (to 150% of the tidal volume delivered by the mechanical ventilator), the use of a slow inspiratory flow rate (achieved by a slow compression of the resuscitation bag), an inspiratory pause (allowing complete distribution of the inflated air among all the ventilated lung parts) and a high peak expiratory flow achieved by the rapid release of the resuscitation bag. It is imaginable that a higher expiratory than inspiratory flow promotes the outwardly directed motion of airway secretions [12,13].

Although mobilization of airway secretions and prevention of sputum plugging [11,14], and improved alveolar recruitment [10] are cited as potential benefits of bagging, the evidence supporting its efficacy is lacking.

We hypothesized MH to benefit cardiac surgery patients. Specifically, we hypothesized MH maneuvers to attenuate the reduction of FRC, to prevent hypoxemia and to prevent atelectasis after cardiac surgery. To test this hypothesis we designed a randomized controlled trial to compare a strategy using frequent MH maneuvers with a strategy only using MH if clinically indicated.

## Materials and methods

### Patients and setting

**Figure 1:**
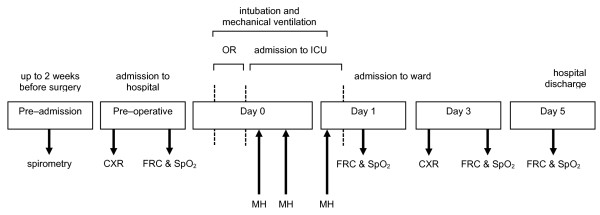
**Time line depicting trial investigations and interventions**. Note that time till tracheal extubation is variable among patients, as is the number of manual hyperinflation maneuvers. Abbreviations: OR, operation room; ICU, intensive care unit; CXR, chest radiography; FRC, functional residual capacity; MH, manual hyperinflation.

This trial was conducted from February 2009 to March 2010 at the 28-bed intensive care unit (ICU) of the Academic Medical Center, Amsterdam, The Netherlands. The Institutional Review Board approved the study. Pre-operative informed consent was obtained from patients scheduled for elective coronary artery bypass graft and/or valve surgery. Figure 1 presents a timeline depicting the trial interventions.

Pre-operative spirometry was performed within two weeks before surgery by trained respiratory technicians according to the latest recommendations [15]. Patients after previous lung surgery or moderate to severe chronic obstructive pulmonary disease (post-bronchodilator forced expired volume in one second (FEV_1_) < 80% predicted and the ratio of forced expiratory volume in one second and forced capacity (FEV_1_/FVC ratio) < 0.7) [16] were excluded from participation.

Patients who required repeated cardiac surgery within 48 hours after the index surgical procedure and patients who required prolonged post-operative mechanical ventilation (> 36 hours) were excluded from the analysis, since it would be impossible to measure FRC in these patients on the predefined time points.

Preoperative risk factors were scored with the European System for Cardiac Operative Risk Evaluation score (the Euroscore), a scoring system used to predict mortality among cardiac surgery patients [17].

### Manual hyperinflation

Attending ICU nurses performed MH maneuvers using a standard technique [18,19]. Preceding this randomized controlled trial, all ICU nurses took part in an educational program that highlighted the several factors considered important in performing MH [20]. MH was performed in the supine position within 30 minutes after connection to the ICU ventilator, repeated every 6 hours and directly before tracheal extubation.

A Mapelson-C circuit with a two liter re-breathing bag (Medicare, Uitgeest, The Netherlands) with the gas flow entry and the expiratory valve located at the patient end of the circuit (a standard bedside resuscitation and manual hyperinflation circuit) was used to perform the maneuver. The circuit was connected to wall oxygen at 15 L/minute and fraction of inspired oxygen (FiO_2_) 1.0. Endotracheal suctioning was performed in sequence with the MH-procedure to remove secretions if mobilized by MH.

### Randomization

On admission to the ICU, patients were randomly assigned to a strategy using frequent MH maneuvers (the "routine MH group") or a strategy using MH only on clinical indication (the "control group"). Randomization was by cards in opaque sealed envelopes drawn by an independent person.

The "routine MH group": A MH maneuver was performed within 30 minutes after connection to the ICU-ventilator, repeated every 6 hours and directly before tracheal extubation. In case of perceptible sputum [21,22] which could not be removed from the larger airways with endotracheal suctioning, or in case of a drop in oxygen saturation (SpO_2_, < 95%), an additional MH maneuver could be performed. It was left to the discretion of the attending ICU nurse whether MH was accompanied by tracheal suction.

The "control group": A MH maneuver was only performed in case of perceptible sputum which could not be removed from the larger airways with endotracheal suctioning, or in case of a drop in SpO_2 _(< 95%).

### Standard procedures

Independent anesthesiologists, ICU physicians and nurses not involved in the study carried out standard procedures.

Patients were anesthetized according to the institutional protocol for cardiac surgery; pre-medication consisted of 1 to 2 mg lorazepam and anesthesia was induced by etomidate, sufentanil, and rocuronium. During the surgical procedure, sufentanil (150 to 250 micrograms) was used as an analgesic, and sevoflurane plus propofol were used to maintain anesthesia. Morphine (5 mg) and midazolam (5 mg) were given at the end of the procedure. The ventilatory protocol consisted of volume-controlled ventilation with a tidal volume of 6 to 8 ml/kg, at an inspired oxygen fraction of 0.40, positive end expiratory pressure of 5 cmH20, inspiratory-to-expiratory ratio of 1:2, and a respiratory rate adjusted to achieve normocapnia. Cardiopulmonary bypass was performed under moderate hypothermia (32°C), using a membrane oxygenator and non-pulsatile blood flow. At the end of anesthesia, all patients were transferred to the ICU with tracheal intubation.

The post-operative ICU protocol involved fluid resuscitation with normal saline and starch solutions, blood transfusion to maintain hemoglobin concentration ≥ 5.0 mmol/L, dopamine and norepinephrine in continuous infusion to achieve mean arterial blood pressure ≥ 70 mmHg, and dobutamine and/or enoximone to achieve a cardiac index ≥ 2.5 L/minute/m^2 ^or a mixed venous oxygenation ≥ 60%.

Sedation and analgesics were titrated according to the ICU protocol for cardiac surgery patients. Neuromuscular blocking drugs were never used in the ICU. Propofol was given for sedation via continuous infusion until the core temperature was ≥ 36.0°C. Acetaminophen (4 g/day) was started in all patients. Attending ICU nurses assessed requirement for analgesia during the entire ICU stay. Morphine was given in boluses of 1 to 2 mg, and repeated as needed.

The trachea was extubated after achieving general tracheal extubation criteria (that is, responsive and cooperative, hemodynamic stability, chest tube drainage < 100 mL last hour, having a rectal temperature > 36.0°C, a respiratory frequency of 10 to 20 breaths/minute without machine-controlled breaths, FiO_2 _of 40%, and a pressure support level of 5 to 10 cmH_2_O for 30 minutes). After extubation oxygen was administered to the nostrils, typically 3 to 5 L/minute. If PaO_2 _was > 100 mmHg (13.3 kPa), administration of oxygen was stepwise reduced to 1 L/minute.

All patients were mobilized early, and had daily routine chest physiotherapy. On the first or second postoperative day, patients were discharged from the ICU and transferred to an intermediate care unit.

### Primary endpoint

The primary endpoint was the change of FRC from the day before cardiac surgery to one, three, and five days after tracheal extubation. FRC measurements were performed with use of the "He re-breathing technique" (Masterscreen-PFT; Jaeger, Wurzberg, Germany) according to the European Respiratory Society recommendations [23]. During FRC measurements, patients were breathing room air while in bed in the upright sitting position. The mean value of 2 technical acceptable measurements was recorded. All measurements were performed by the same investigator recorded who was blinded for randomization groups.

### Secondary endpoints

Pulse-oximeter oxygen saturation (SpO_2_) was measured with commercial pulse- oximeters (IntelliVue pulse-oxymetrie; Philips, Eindhoven, The Netherlands; or Datascope 'Duo' with Masimo SET^®^, Hoevelaken, The Netherlands) during the first five days after tracheal extubation. SpO_2 _was measured for 10 minutes while the patient was breathing room air. Measurements were terminated when SpO_2 _dropped to a level ≤90%. The latter was defined as hypoxemia, and supplemental oxygen therapy was continued with its occurrence.

Chest radiographs were obtained in a sitting position, both pre-operative and on the third post-operative day. An independent radiologist blinded for randomization groups interpreted the chest radiographs. Each radiograph was evaluated for the presence of atelectasis, pulmonary infiltrate, extra-vascular lung fluid, pleural fluid, and pneumothorax. The presence of atelectasis was graded as: no atelectasis ("0"); plate or sub-segmental atelectasis ("1"); segmental atelectasis ("2"); or lobair atelectasis ("3"); for the purpose of this study, extent of other abnormalities was graded as: not present ("0"); mild ("1"); moderate ("2"); or severe ("3").

### Other data collected

These included the number of MH maneuvers per patient; demographics (age, gender, body weight, body height, type of surgery), duration of anesthesia, anesthetics given to the patient during surgery, duration until tracheal extubation and length of stay in ICU and hospital, propofol and morphine dose in the ICU.

### Power calculation

This trial was designed to detect a 300 mL difference in the post-operative FRC between the two study groups (10). A sample size of 42 in each group would have 80% power to detect a difference in means of -300 mL (the difference between a Group 1 mean, of 1.900 mL and a Group 2 mean, of 2.200 mL), assuming that the FRC standard deviation was 480 mL using analysis of variance (ANOVA) for repeated measurements with a 0.05 two-sided significance level. Based on an anticipated drop-out of 15 to 20% we randomized a total of 100 patients.

### Statistical analysis

Data were presented as mean with standard deviation (SD) or medians with interquartile range (IQR) where appropriate. Descriptive statistics were used to summarize patient characteristics. Differences in FRC and SpO_2 _at the different time points were compared by analysis of variance (ANOVA) for repeated measurements. Categorical variables (hypoxic periods, chest radiograph data and MH data) were compared between groups by X^2 ^tests. All participants were analyzed in the groups that they were allocated to. The level of significance was set at 0.05. All analyses were performed using SPSS for Windows (version 18.0, SPSS Inc., Chicago, IL, USA).

## Results

**Figure 2:**
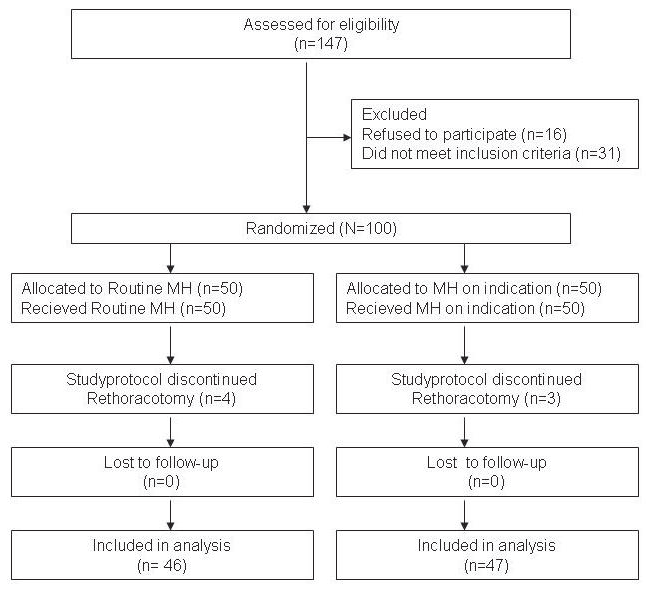
**CONSORT diagram of the trial**.

One hundred patients were randomly assigned to one of the two groups (Figure 2). Seven patients (four in the routine MH group and three in the control group) were not analyzed because they needed repeated cardiac surgery. In nine patients (six in the routine MH group and three in the control group) FRC measurements could not be performed on predefined time points because of tracheal extubation > 48 hours or re-intubation for cardiac instability (10). In three other patients, (one in the routine MH group and two in the control group), FRC measurements could not be performed on predefined time points because of tracheal extubation > 48 hours because of respiratory failure (2).

Two patients died; one patient in the routine MH group died of bowel ischemia after four days; one patient in the control group died due to cardiac failure.

Groups were comparable for all demographic data (Table 1). No differences were found for time till tracheal extubation or stay in ICU or hospital. Administered analgesics, sedatives and blood products were similar between groups. Ventilator data are given in Table 2

**Table 1:** Characteristics of the study population

	Routine MH (N = 50)	Control (N = 50)
*Demographics*		
Age, yrs	65 ± 10.2	60.3 ± 13.5
Male, n	35	38
BMI, kg/m^2^	25.8 ± 4.2	26.7 ± 4.7
FEV1, %	95 ± 14	99 ± 16
FVC, %	101 ± 14	101 ± 15
EuroScore, %	5.4 ± 2.7	4.4 ± 2.8
Current smokers, n	14	10
*Type of surgery*		
CABG, n	13	16
Valve surgery, n	29	24
Combined CABG/valve surgery, n	8	10
LIMA, n	20	22
*Intra-operative and intensive care characteristics *		
Duration of surgery, minutes	305 ± 84	300 ± 73
CPB time, minutes	134 ± 58	128 ± 61
Total sufentanil dose, microgram	188 (143 to 250)	250 (150 to 250)
Total midazolam dose, mg	15 (9 to 20)	15 (5 to 25)
Total morphine dose, mg	21 (9 to 20)	15 (5 to 25)
Total propofol dose, mg	1.250 (785 to 1.800)	1.478 (1.060 to 2.010)
WBC on ICU admission (× 10^6^)	10 ± 3	10 ± 3
Core temperature on ICU admission (°C)	35.7 ± 0.8	35.6 ± 0.9
Sedation duration (ICU), hours	3.9 (2.0 to 13.9)	3.5 (2.3 to 18.0)
Duration of tracheal intubation (ICU), hours	10.5 (6.5 to 16.0)	10.8 (7.3 to 24.6)
Length of stay (ICU), hours	23.2 (20.8 to 44.8)	23.2 (20.7 to 46.3)
Length of stay (hospital), days	5.3 (4.3 to 7.4)	5.4 (4.4 to 7.2)

Data are presented as mean ± SD or median (IQR).

Abbreviations: BMI, body mass index; CABG, coronary artery bypass graft; FEV1, forced expired volume in one second, expressed as percentage of predicted value; FVC, vital capacity, expressed as percentage of predicted value; EuroScore, based on system predicting mortality with cardiac surgery (%); CPB, cardio pulmonary bypass; LIMA, left internal mammary artery; WBC, white blood cell count.

**Table 2:** Respiratory data

		Routine MH (N = 46)	Control (N = 47)	*P*-value
PEEP (cmH2O)	OR	5 ± 0.1	5 ± 0.1	*P *= 0.90
	ICU	5 ± 1	6 ± 2	*P *= 0.20
PIP (cmH_2_O)	OR	16 ± 3	16 ± 3	*P *= 0.70
	ICU	17 ± 3	18 ± 3	*P *= 0.15
Tidal volume (ml/kg IBW)	OR	7.7 ±.1.2	7.4 ± 1.2	*P *= 0.20
	ICU	7.9 ± 1.3	7.9 ± 1.3	*P *= 0.82
Maximum FiO_2_	OR	0.85 ± 0.17	0.82 ± 0.19	*P *= 0.43
	ICU	0.53 ± 0.9	0.50 ± 0.17	*P *= 0.28
FiO_2_	OR	0.48 ± 0.12	0.48 ± 0.12	*P *= 0.87
	ICU	0.47 ± 0.09	0.47 ± 0.07	*P *= 0.97

Data are presented as mean ± SD.

Abbreviations: PEEP, Positive End Expiratory Pressure; OR, Operating Room; ICU, Intensive Care Unit; PIP, Positive Inspiratory Pressure; IBW, Ideal Body weight; FiO_2_, Fraction of inspired oxygen.

### MH maneuvers

Patients in the routine MH group received median (IQR) 2 (2 to 3) MH maneuvers compared to 0 (0 to 0) in the control group (*P *< 0.001). Endotracheal suction was performed 2.5 (2 to 4) times in the patients in the routine MH group compared to 1 (1 to 2) in the patients in the control group (*P *< 0.001).

### Changes in FRC

**Figure 3:**
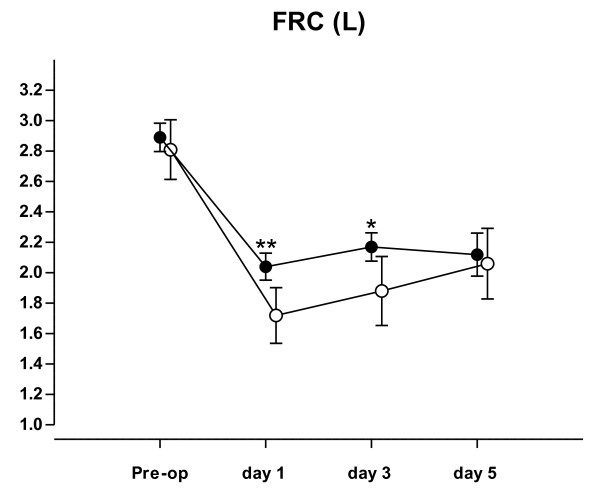
**Measurements of functional residual capacity**. Preoperative functional residual capacity (FRC (L) mean 95% CI) and FRC at one, three and five days after tracheal extubation in routine MH (N = 37; closed symbols) and control group (N = 41; open symbols). *, *P *< 0.04, **, *P *< 0.002.

In both groups, FRC was significantly reduced after cardiac surgery (from 2.840 ml ± 720 to 1,815 ml ± 540 ml, difference = 1.025 ml, 95% CI 877 to 1,176 ml, *P *< 0.001) (Figure 3).

In the routine MH group FRC decreased on the first post operative day to 71% of the pre-operative value (from 2.875 ± 565 mL to 2.055 ± 540 mL), while in the control group FRC decreased to 57% of pre-operative level (from 2.808 ± 650 mL to 1.588 ± 530 mL). The difference was 400 mL, 95% CI 680 to 115 mL, *P *= 0.002).

The absolute reduction in FRC at Day 3 was lower in the routine MH strategy group (750 ± 565 mL) compared to the control group (1,056 ± 570 mL) (difference = 306 mL, 95% CI 593 to 20 mL *P *= 0.04). Differences in decreases of FRC between the two study groups were no longer statistical significant at Day 5 (760 ± 500 mL in the routine MH group and 730 ± 530 mL and the control group, respectively).

### Oxygenation

There were no differences between groups in PaO_2_/FiO_2_, either within one hour after admission to the ICU or during intubation and mechanical ventilation. Hypoxemia on the first post-operative day occurred in 17% of the patients in the MH group versus 28% of the patients in the control group, at Day 3 in 13% in the MH group versus 21% in the control group. At Day 5, hypoxemia occurred in 8% of the patients in both groups. Differences did not reach statistical significance (Table 3).

**Table 3:** Clinical outcome data

	routine MH group (N = 46)	control group (N = 47)	*P*-value
First PaO_2_/FiO_2 _(one hour after admission) (mmHg)	339 ± 127	329 ± 126	*P *= 0.70
Worst PaO_2_/FiO_2_, (while intubated and mechanically ventilated) (mmHg)	213 ± 70	221 ± 82	*P *= 0.60
Hypoxemia - Day 1 (n (%))	8 (17)	13 (28)	*P *= 0.23
Hypoxemia - Day 3 (n (%))	6 (13)	10 (21)	*P *= 0.29
Hypoxemia - Day 5 (n (%))	3 (8)	3 (8)	*P *= 0.60
SpO_2 _- Day 1 (%)	95 (93 to 97)	94 (90 to 97)	*P *= 0.75
SpO_2 _- Day 3 (%)	95 (91 to 96)	94 (92 to 96)	*P *= 0.75
SpO_2 _- Day 5 (%)	96 (94 to 97)	96 (95 to 97)	*P *= 0.75

Data are presented as percentages of N (%), medians (IQR) or means ± SD. Hypoxemia was defined as a drop of SpO_2 _≤90% within five minutes after cessation of oxygen supply.

Abbreviations: SpO_2_, peripheral oxygen saturation; PaO_2_, partial pressure of arterial oxygen; FiO_2_, fraction of inspired oxygen.

### Chest radiographs

Pre-operative chest radiographs showed no atelectasis. At Day 3 more patients in the control group had atelectasis compared to patients in the routine MH strategy group (Table 4). However, there was no difference in the atelectasis score between the two groups. Pneumothorax was found in two patients, one in each study group. A mild pulmonary infiltrate was found in one patient in the routine MH group; moderate pulmonary infiltrates were found in two patients, one in each study group.

**Table 4:** Chest radiograph results

	routine MH group (N = 46)	control group (N = 47)	*P*-value
No atelectasis	8 (17)	0 (0)	*P *= 0.002
Plate/subsegmental atelectasis	20 (44)	22 (47)	*P *= 0.10
Segmental atelectasis (one of two segments)	18 (39)	23 (49)	*P *= 0.55
Lobar atelectasis	0 (0)	2 (4)	*P *= 0.16

Data are presented as percentages of N (%)

## Discussion

The results of our randomized controlled trial can be summarized as follows: (1) FRC is significantly reduced in patients after cardiac surgery for several days; (2) MH partly prevents this reduction; (3) the incidence of signs of atelectasis on chest radiographs differed between groups.

Our study is the first randomized controlled trial assessing the effects of MH on FRC in patients after cardiac surgery. Its findings are in line with the results of other studies suggesting that MH may benefit patients after surgery. A previous randomized controlled trial showed early MH to improve PaO_2 _and static compliance in patients after myocardial re-vascularisation [24]. Also, MH reduced duration of mechanical ventilation in that trial. Another randomized controlled trial confirmed MH to improve oxygenation after surgery [25].

The reduction of FRC and the presence of atelectasis after cardiac surgery in our trial are consistent with findings in previously published studies of cardiac surgery patients. In one study deterioration of pulmonary function was frequently seen after cardiac surgery, with FRC reductions of 40 to 50% during the first 24 hours after tracheal extubation [26]. Also, incidence of signs of atelectasis on chest radiographs occurred in up to 90% of patients after cardiac surgery, and its presence was associated with a larger decrement in pulmonary function. In another study, atelectasis persisted for several days after surgery, was likely to be a focus of infection and could have been a contributing factor to pulmonary complications [27].

Plugging of airway secretions may lead to airway obstructions, thereby causing gas resorption distal to the obstructions in patients who are mechanically ventilated. Beneficial effects of MH on clearance of airway secretion have been demonstrated before [14]. Higher expiratory than inspiratory flows generated with MH is the proposed mechanisms that contribute to clearance of airway secretions in intubated and mechanically ventilated patients. In accordance, in our study endotracheal suction was performed more often in patients in the routine group compared to patients who received on demand MH, suggesting improved clearance of secretions necessitating airway suctioning. Unfortunately, the amount and appearance of eliminated secretions were not compared between groups, which could be seen as a limitation of our trial.

Our data suggest that MH could contribute to the recovery of collapsed lung areas after cardiac surgery. Delivering increased tidal volumes with an inspiratory pause via MH could generate transpulmonary pressures that could overcome, at least in part, alveolar collapses [28]. Earlier studies concluded that for complete reopening of collapsed lung tissue inspirations are required that reach airway pressures of 40 cmH_2_O which are maintained for eight to nine seconds [29]. Certainly, MH is not capable of providing such pressures for longer than a few seconds. However, short-lasting natural occurring maneuvers, such as coughing and sighs, also result in recruiting of lung tissue.

Hypoxemia occurred in 23% of patients at Day 1 after tracheal extubation. These findings were in contrast to previous studies, which report up to 60% of patients with hypoxic periods within the first two days after tracheal extubation [26,30]. There are several possible explanations as to why MH in our trial did not result in a greater difference in the presence of hypoxemia. One reason could be that oxygenation not only results from changes in (regional) ventilation but also from changes in (regional) perfusion. Hypoxic pulmonary vasoconstriction may be responsible for maintaining the ventilation-perfusion ratio during localized alveolar hypoxia. Thus, if hypoxic pulmonary vasoconstriction works, oxygenation is preserved even during atelectasis. This could, at least in part, explain our findings. Another reason could have been the use of the pulse-oximeter to detect hypoxemia. Although the pulse-oximeter is an device with a reasonable degree of accuracy, the performance of the pulse-oximeter can be disrupted by several, including poor peripheral perfusion and peripheral vasoconstriction [31]. We cannot exclude the possibility that the pulse-oximeter was not discriminative enough. It may be more plausible that our trial was insufficiently powered to detect a difference in hypoxemia between groups.

The incidence of signs of atelectasis on chest radiographs differed between groups. Indeed, significantly fewer patients in the routine MH group had radiographic signs of atelectasis. Reasons for atelectasis after cardiac surgery are multiple and include higher levels of intra-operative FiO_2_, type of surgery, obesity and post-operative pain. Absorption atelectasis during mechanical ventilation is most likely to occur when levels of FiO_2 _are high [27]. Indeed, higher levels of FiO_2 _during mechanical ventilation because of general anesthesia during surgery are associated with atelectasis formation [27,32]. Another causative factor could be the harvesting of the internal mammary artery [33]. Use of the internal mammary artery graft has been found to be associated with a greater occurrence of post-operative atelectasis. Finally, post-operative pain leads to impairment of deep breaths and, therefore, could enhance formation of atelectasis [34]. The differences between groups could not be explained by the above-mentioned factors, since they affected both groups to the same extent.

MH may cause a short-term state of hyperinflation and as such it can be questioned if MH is a safe procedure. We, and others, have studied possible adverse effects of MH [19,35,36]. These studies suggested that MH, when performed under controlled conditions and/or performed by experienced and trained nurses, has negligible side-effects. In our previous experiences we neither registered side-effects prospectively nor did we evaluate patient comfort in the present trial.

We excluded patients who required prolonged post-operative mechanical ventilation (> 36 hours) from analysis of the primary endpoint, since it was impossible to measure FRC in these patients on the predefined time points. We explicitly intended to study post-extubation changes in FRC, since FRC is affected by intubation and mechanically ventilation, especially when positive end expiratory pressure (PEEP) is applied (as was the case for all these patients). The patients, who were excluded from the analysis of the primary endpoint, were analyzed for secondary endpoints. The analysis of the secondary endpoints supports the analysis of the primary endpoint.

We recognize several other limitations of our study. First, the bedside FRC measurement is not the most optimal technique for quantifying lung aeration. Frequent computer tomography scanning, considered the gold standard for quantifying lung aeration, however, could not be performed for obvious reasons [37]. Nevertheless, recently bedside FRC measurement techniques have been evaluated with acceptable accuracy and repeatability [38]. In addition, in another study changes in FRC after alveolar de- and re-recruitment could be easily assessed [39]. A second limitation is that we did not measure MH parameters considered important for its efficacy. However, we recently showed that the implementation of MH guidelines, guided by individual feedback and educational meetings led to consistent MH performance [20]. A third limitation considers generalizability of our findings. Our study was a single-center trial, and practice may differ elsewhere. For instance, tracheal intubation times in our cohort are different from those reported elsewhere.

## Conclusions

MH partly prevents reduction of FRC in patients after cardiac surgery in the first post-operative days. In accordance, the incidence of signs of atelectasis on post-operative chest radiographs is significant lower in patients who receive MH. Future studies are needed to determine the effect of MH on important clinical endpoints, including duration of tracheal intubation, post-operative pulmonary complications and duration of hospitalization.

## Key messages

• In patients after cardiac surgery, reduction of FRC is partly prevented by manual hyperinflation.

• The incidence of signs of atelectasis on post-operative chest radiographs in patients after cardiac surgery is lower in patients who receive manual hyperinflation.

## Abbreviations

ANOVA: analysis of variance; BMI: body mass index; CABG: coronary artery bypass graft; CXR: chest radiography; CPB: cardio pulmonary bypass; FEV1: forced expired volume in one second, expressed as percentage of predicted value; FEV1/FVC: ratio of forced expiratory volume in one second and forced capacity; FiO_2_: fraction of inspired oxygen; FRC: functional residual capacity; FVC: forced vital capacity, expressed as percentage of predicted value; IBW: ideal body weight; ICU: intensive care unit; IQR: interquartile range; LIMA: left internal mammary artery; MH: manual hyperinflation; OR: operating room; PaO_2_: partial pressure of arterial oxygen; PaO_2_/FiO_2_: ratio of partial pressure of arterial oxygen and fraction of inspired oxygen; PEEP: positive end expiratory pressure; PIP: positive inspiratory pressure; SD: standard deviation; SpO_2_: peripheral oxygen saturation; WBC: white blood cell count.

## Competing interests

The authors declare that they have no competing interests.

## Authors' contributions

FP contributed to the conception and design of the trial, data collection, statistical analysis, and writing of the manuscript. DPV contributed to data collection, and the revision of the manuscript. SBdeN contributed to the study design, data collection and the revision of the manuscript. LFB contributed to the data collection, and the revision of the manuscript. PB contributed to the study design and the revision of the manuscript. BAdeM contributed to the study design and the revision of the manuscript. JMB contributed to the conception and design of the trial, statistical analysis and writing of the manuscript. MJS contributed to the conception and design of the trial, interpretation of the data and writing of the manuscript. All authors read and approved the final version of the manuscript.
